# Novel structural aspect of the diatom thylakoid membrane: lateral segregation of photosystem I under red-enhanced illumination

**DOI:** 10.1038/srep25583

**Published:** 2016-05-05

**Authors:** David Bína, Miroslava Herbstová, Zdenko Gardian, František Vácha, Radek Litvín

**Affiliations:** 1 Institute of Plant Molecular Biology, Biology Centre CAS, Department of Photosynthesis, Branišovská 31, České Budějovice, 37005, Czech Republic; 2 Faculty of Science, University of South Bohemia, Institute of Chemistry and Biochemistry, Branišovská 1760, České Budějovice, 37005, Czech Republic

## Abstract

Spatial segregation of photosystems in the thylakoid membrane (lateral heterogeneity) observed in plants and in the green algae is usually considered to be absent in photoautotrophs possessing secondary plastids, such as diatoms. Contrary to this assumption, here we show that thylakoid membranes in the chloroplast of a marine diatom, *Phaeodactylum tricornutum*, contain large areas occupied exclusively by a supercomplex of photosystem I (PSI) and its associated Lhcr antenna. These membrane areas, hundreds of nanometers in size, comprise hundreds of tightly packed PSI-antenna complexes while lacking other components of the photosynthetic electron transport chain. Analyses of the spatial distribution of the PSI-Lhcr complexes have indicated elliptical particles, each 14 × 17 nm in diameter. On larger scales, the red-enhanced illumination exerts a significant effect on the ultrastructure of chloroplasts, creating superstacks of tens of thylakoid membranes.

Integral membrane proteins form a cornerstone of natural energy conversion machinery. This applies also to the complexes carrying out the primary processes of photosynthesis in the thylakoid membrane: the light-harvesting systems (antennas) that capture the incident radiation, and the complexes that perform the charge separation and subsequent electron transfer steps, photosystems and cytochrome b_6_/f complex. The electron transfer between individual membrane complexes is mediated by diffusible carriers moving both inside the hydrophobic core of the lipid bilayer and along its hydrophilic surface.

Proper supramolecular organization is crucial to maintain high efficiency of the transfer processes. The efficient excitation energy transfer requires (i) an accumulation of light-absorbing pigments and (ii) a close association of the antenna complexes and reaction centres (RCs)^1^. On the other hand, diffusion-dependent movement of molecules through the membrane can be restricted by a high protein packing density^2^. Moreover, the supramolecular organization of the thylakoid membrane has to respond to the changing environmental conditions and, in turn, the structural changes control the diffusion-dependent processes such as electron transfer and/or molecular repair^3^. Knowledge of the spatial arrangement of the supercomplexes in intact photosynthetic membranes is therefore essential for understanding the function of this highly composite and dynamic system.

Among eukaryotes, the overall thylakoid membrane architecture and supramolecular organization of the photosynthetic machinery is best studied in plants. The internal thylakoid membrane system of the plant chloroplast is differentiated into appressed (grana) and unappressed (stroma lamellae) regions, to which photosystems (PSI and PSII) are spatially segregated. This arrangement is termed lateral heterogeneity^4^. Typically, PSII and its light-harvesting system (LHC) II reside in the grana stacks, whereas PSI-LHCI complexes along with ATP synthase are sterically excluded from the grana core and localized to the stroma-facing lamellae. According to an evolutionary hypothesis, plants have evolved grana after land colonization when larger light-harvesting antennas became advantageous for adaptation to shade^5^. Under low-light conditions, the light harvesting capacity is enhanced through the formation of the highly ordered semicrystalline arrays of PSII-LHCII supercomplexes^6^,^7^,^8^. A similar degree of organization has not been reported for PSI-LHCI supercomplexes.

Relative to plants, whose thylakoid membranes show extreme lateral heterogeneity, much less is presently known about the supramolecular organization of the pigment-protein complexes in the thylakoid membranes of other photosynthetic eukaryotes. In chloroplasts of green algae, the primary endosymbiotic organisms that gave rise to plants, thylakoids have appressed and unappressed regions but not as highly structured as in plants. Thus, a certain degree of lateral heterogeneity is observed in some species of green algae^9^,^10^,^11^. However, it is generally accepted that the extent of photosystem segregation is very limited in algae bearing a secondary plastid. The stramenopile thylakoids typically form extended stacks of three loosely appressed membranes running in parallel throughout the plastids^12^. Moderate differences in the photosystem localization have been proposed for diatom thylakoids, with the outer lamellae of stacks being enriched in PSI and ATP synthase compared to the inner parts of the stacks^13^,^14^. On the contrary, evidence for a certain degree of lateral heterogeneity of distribution of PSI (but not PSII), not in relation to appressed and unappressed membranes, exists for stramenopile groups other that diatoms^15^.

At the molecular level, in diatoms, available data do not support the existence of large PSII-antenna supercomplexes, a fact that could be likely accounted for by the lack of small chlorophyll protein (CP) subunits of PSII^16^. On the other hand, stable antenna-PSI associations have been reported, and it appears likely that there exists a PSI-specific pool of antenna complexes^16^,^17^,^18^,^19^. A PSI-antenna association based on a monomeric PSI core with an asymmetrically placed row (or two rows) of antenna units appears to be a blueprint common to all eukaryotic phototrophs^20^,^21^,^22^,^23^.

Diatoms have been extensively studied as a key taxon with great species diversity, prominent in marine ecosystems. Knowledge of the molecular biology of diatoms has advanced in the recent years, being spurred by the sequencing of complete genomes. However, despite the global ecological importance of diatoms as primary producers^24^,^25^,^26^ and their remarkable adaptability^17^, understanding the molecular architecture and dynamics of the thylakoid membranes, and of their functional significance for the photosynthetic machinery, is still inadequate. Although earlier studies^13^,^14^,^27^,^28^ support the view that PSII and PSI complexes are for most part randomly distributed in the thylakoids of stramenopile species including diatoms, we hypothesize that under specific environmental conditions, the segregation of photosystems could have evolved at least in some secondary endosymbiotic algae.

The present study has focused on the supramolecular organization of the thylakoid membranes in the secondary plastid of the marine diatom *P. tricornutum* grown under low light, enhanced in the red/far red component. The resulting phenotype is characterized not only by high plasticity of the light harvesting system but also by lateral segregation of the photosystems. Data presented here suggest a hitherto unknown structural plasticity of the stramenopile photosynthetic membrane. We propose that the force driving the segregation process in the diatoms is similar to that forming the grana in plants, a necessity of adaptation for shaded environments.

## Results

**Figure 1:**
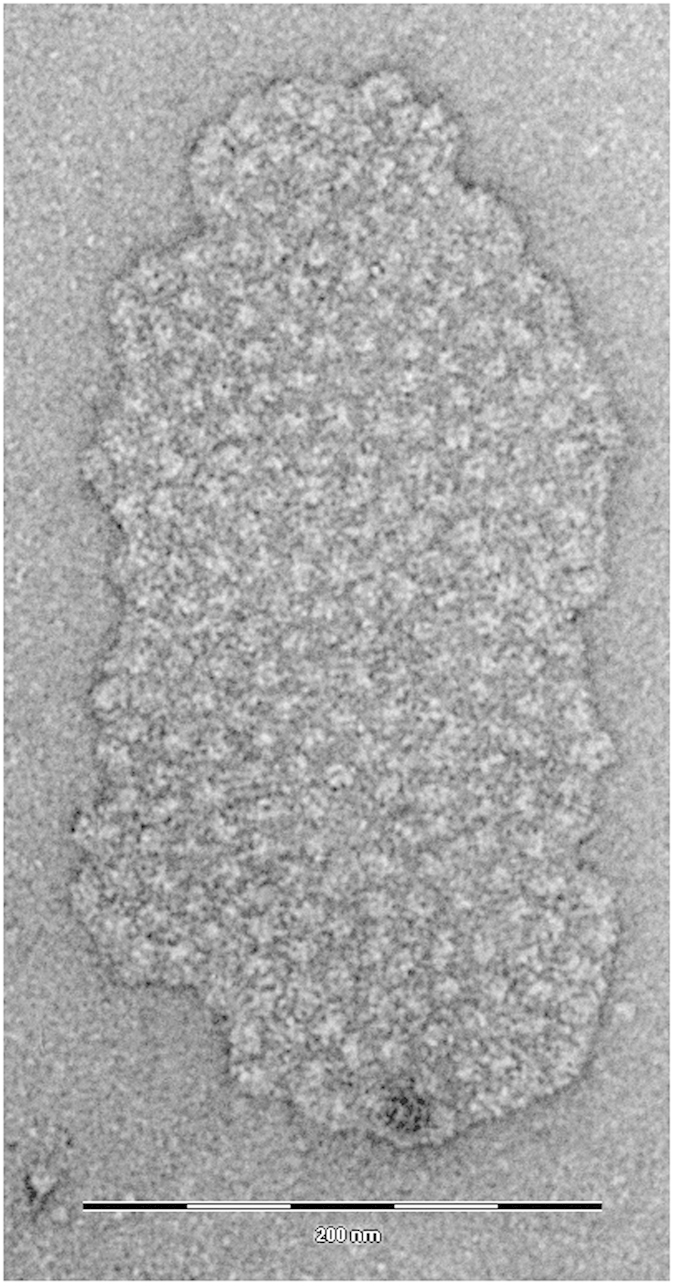
An example of an intact membrane patch isolated from thylakoid membranes of *P. tricornutum* grown under red-enhanced illumination. Scale bar corresponds to 200 nm.

A mild solubilization of the thylakoid membranes of red-light (RL)-adapted *P. tricornutum* and subsequent purification steps yielded a fraction containing large membrane patches as seen in Fig. 1. The first eluted fraction from a size-exclusion chromatography column (Supplementary Fig. S1) consisted of flat membrane sheets covered quite evenly with weakly stained particles (see Supplementary Fig. S2 for more examples). Such a fraction was absent in analyses of cultures grown under simulated day-light conditions^17^ (see Suplementary Fig. S1c). The area of individual membrane patches observed in our samples ranged from 2400 nm^2^ to 65000 nm^2^ (mean 24300 nm^2^). The number of particles per patch scaled linearly with patch area, yielding mean particle density of 4600 ± 200 particles per square micrometre of the membrane. As seen in Figs 1 and S2, the observed patches were homogeneous in appearance. There was no indication of presence of surrounding membrane or a transition between regions occupied with different types of particles corresponding to features observed in e.g. freeze fracture studies^15^. Therefore, the native size of these regions cannot be straightforwardly estimated from present data. These patches, thus, could have been broken off from larger membrane sheets or the surrounding membranes might have been removed by the detergent treatment. Partial detergent extraction of the protein complexes from the edges of the patches could have also occurred.

### Membrane domains formed by Photosystem I with Lhcr antenna

**Figure 2:**
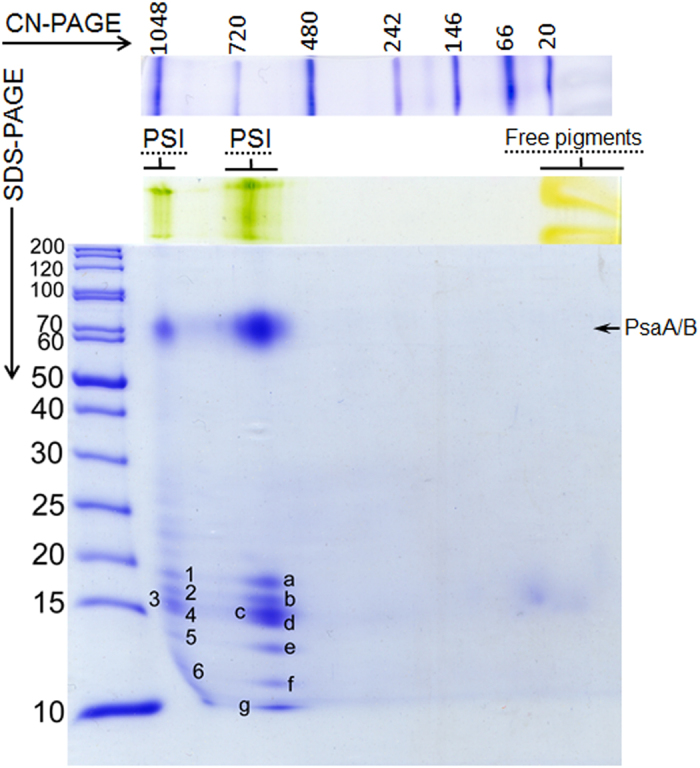
Protein composition analysis of the membrane patches isolated from red-light grown *P. tricornutum*. Gels are representative of three replicates.

As seen in Fig. 2, the protein composition of the membrane patches was simple, showing exclusively PSI and its antenna system formed by a set of the Lhcr proteins. A clear native polyacrylamide gel electrophoresis (CN-PAGE) of fraction 1 yielded green bands of about 1050 kDa and 700 kDa with similar protein composition, as seen in the second dimension SDS-PAGE (Fig. 2). MS/MS protein analysis of spots marked *1–6* (CN-1050) and *a–g* (CN-700) in the Fig. 2 identified small subunits of PSI (PsaF, PsaL, PsaD, PsaE) and a large number of Lhcr antenna proteins, namely Lhcr 1–4, 12, 13, 14 (see Supplementary Table 1 for complete results). Lack of brownish protein bands of lower molecular masses in the CN-PAGE around 66 kDa (Fig. 2) suggested the absence of (trimeric) fucoxanthin-chlorophyll *a*/*c* binding protein (FCP) complexes consisting of Lhcf proteins. However, as indicated by a faint spot below 20 kDa in the second dimension SDS-PAGE gel, the fraction 1 contained a trace amount of free protein, possibly monomeric FCPs running together with the front of the free pigments (CN-PAGE around 20 kDa). The fractions eluted from gel filtration column were also tested for the presence of other components of the photosynthetic machinery, i.e. PSII and cytochromes. Immunoblotting against PSII D1 core protein indicated that fraction 1 contained membrane rafts completely free of PSII (Supplementary Fig. S3B). In-gel heme staining was also applied to the samples with the negative result in fraction 1 indicating the absence of cytochrome b_6_/f complex (Supplementary Fig. S3C).

### Pigment stoichiomentry

**Figure 3:**
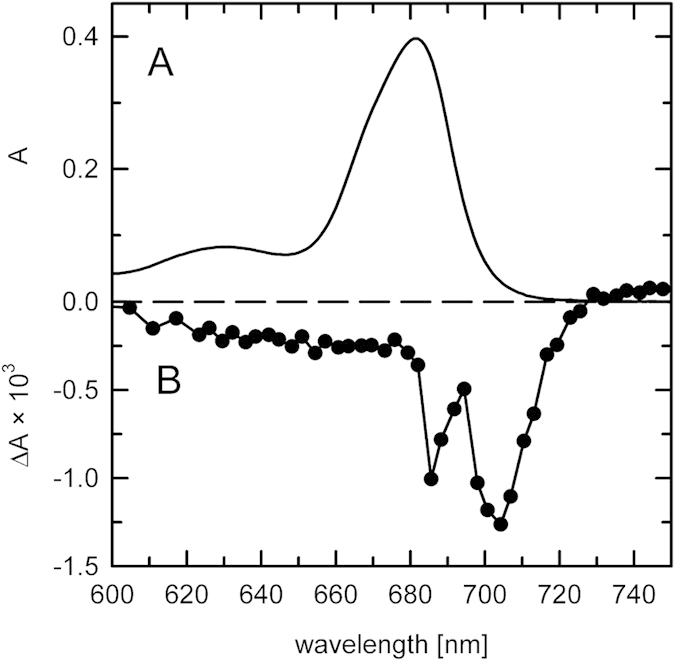
Absorbance spectra of PSI-only membrane patches from *P. tricornutum*. (**A**) – Absorption spectrum, Q_y_ maximum at 680 nm; (**B**) – Flash-induced absorbance difference spectrum, in presence of 20 μM PMS and 20 mM ascorbate. Maximum of bleaching was at 704 nm.

The pigment content of the membrane patches purified by gel filtration was determined by HPLC analysis and the concentration of P700 was estimated using amplitude of photo-induced bleaching of the primary donor absorption in the presence of ascorbate/PMS (20 mM/20 μM) system. The light-induced difference spectrum, shown for convenience in Fig. 3, served as an independent confirmation of the presence of active PSI RC. Moreover, the time constant of re-reduction of photooxidized P700 was found to be 17 ± 2 ms (not shown), in an excellent agreement with published values^29^. The pigment analysis is summarized in Table 1. A ratio of 233 ± 14 chlorophyll (Chl) *a* per one P700 was calculated. This number could be validated independently based on HPLC data only, with the assumption of PSI RC binding of 22 β-carotenes^19^,^30^. Such a value is in reasonable agreement with earlier analyses of diatom and red-algal PSI-antenna assemblies^18^,^19^,^22^. However, the similarity between the present result and the earlier works on diatoms ended with pigments other than Chl *a*. Fucoxanthin: diadinoxanthin ratio of our sample (~1.2:1) was quite different from previous estimates in *P. tricornutum* (~20:1)^18^, interestingly it was reminiscent of data from centric diatoms^19^,^31^. However, the most significant difference from the published reports was found in the amount of Chl *c*. Previous results from both pennate and centric diatom arrived at the Chl *a*:Chl *c* ratio of roughly 10:1, whereas in our samples this number was close to 80:1 (Table 1).

### Spatial distribution of PSI-Lhcr complexes

A rough estimate of area belonging to each particle could be derived from the mean particle density, if the spacing of the visible particles stemmed from a presence of a large part of the complex buried in the membrane. Assuming tight packing and homogeneous size of complexes, 4600 ± 200 particles per 1 μm^2^ (see above) correspond to a particle with an area of 10^6^/4600 (±200) ≈ 220 (±10) nm^2^, that is ~17 nm across, assuming a circular particle. This value is comparable to electron microscopic analyses of PSI-Lhcr complexes from red-algae^20^,^22^ which suggested that in this case the PSI core was accompanied by a semicircular row of Lhcr subunits, yielding elliptical particles with small eccentricity, with axes of about 14 and 17 nm. It is also similar to observation of Veith and Büchel^18^, who reported a diatom PSI-antenna particle, of about 18–20 nm in diameter, although in the cited work no particle analysis was performed, allowing only rough estimation of dimensions.

As seen in Fig. 1, the average apparent size of the particles protruding above the membrane plane, calculated as the full width at half maximum (FWHM) parameter, was less than 10 nm, much smaller than the expected dimension of either the PSI-antenna complex or even the PSI RC core. However, one could assume that the staining of the PSI particles would be uneven and a part of the complex buried in the membrane was not visible in the negatively stained images. A major part of the PSI complex consists of transmembrane helical proteins except for a ridge on top of the stromal side, running approximately perpendicular to the long axis of the PSI RC projection. This surface feature consists of iron-sulfur cluster-binding subunits Psa C, D and E protruding about 3 nm above the plane of the membrane^30^,^32^. This is the part of the complex that could be expected to show on the electron microscopy images, whereas the staining of the membrane part of the complex will likely be hardly distinguishable from the surrounding membrane.

**Figure 4:**
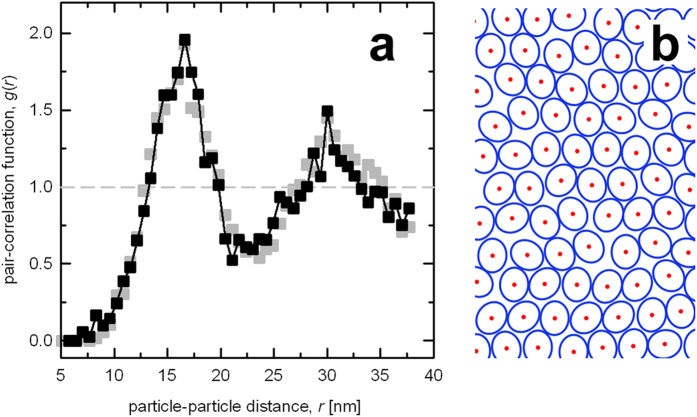
(**a**) Black – experimental pair-correlation function (pcf) computed from the positions of ~1500 particles occupying the total membrane area of 0.3 μm^2^ of 11 separate membrane patches; grey – the pcf computed from simulation of distribution of 1500 elliptical particles of axes 14 and 17 nm representing the PSI-Lhcr complex. A section of a simulated PSI-Lhcr patch is shown in panel b.

In order to gain further insight into the organization of particles in the PSI-rich patches, a spatial analysis was performed. The pair-correlation function (pcf) *g*(*r*), (Fig. 4), clearly demonstrated an order in the positioning of particles, as shown by the distribution function showing maxima (*g*(*r*) > 1) separated by distances of diminished occupancy (*g*(*r*) < 1). Note that *g*(*r*) in the case of a random distribution of particle distances equals 1 (grey dashed line in Fig. 4). The shape of pcf suggests a dense packing of the particles: when one considers an ideal hexagonal lattice with a coordination number of 6, then for a nearest neighbour distance of *r*_1_, the second peak on *g*(*r*) would occur at *r*_2_ = 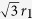 (Supplementary Fig. S4), i.e. for a tightly packed circular particle of 17 nm in diameter, *r*_1_ = 17, *r*_2_ = 29.4, a reasonably close approximation of the observed values, as seen in Fig. 4, even though the packing of particles in the membrane patches was clearly not perfectly hexagonal (Fig. 1). However, considering the estimate of 17 nm obtained from the pcf and the reported dimensions 14 × 17 nm of Lhcr-containing PSI particles found in red algae^20^,^22^ one could establish a working hypothesis of PSI-antenna complex of *P. tricornutum* having the same dimensions. Such hypothesis could be put to test by means of a simulation of the packing of particles of respective size (Fig. 4b) and comparing the resulting pcf with the experimentally observed one.

Since only the spatial distribution of the particles was studied, the PSI-antenna complexes were modelled as hard, noninteracting elliptical discs performing a random walk over a finite-size enclosure (see Methods for details). Ten simulation runs were performed and analysed in the same manner as the native membranes. The resulting pcf of about 1500 particles of the simulated data set is shown in Fig. 4 (*grey symbols*). It clearly showed that packing of ellipses with axes of 14 and 17 nm provided a satisfactory model for the particles occupying the membrane patches isolated from thylakoids of *P. tricornutum*.

### Effect of red light on the thylakoid ultrastructure

**Figure 5:**
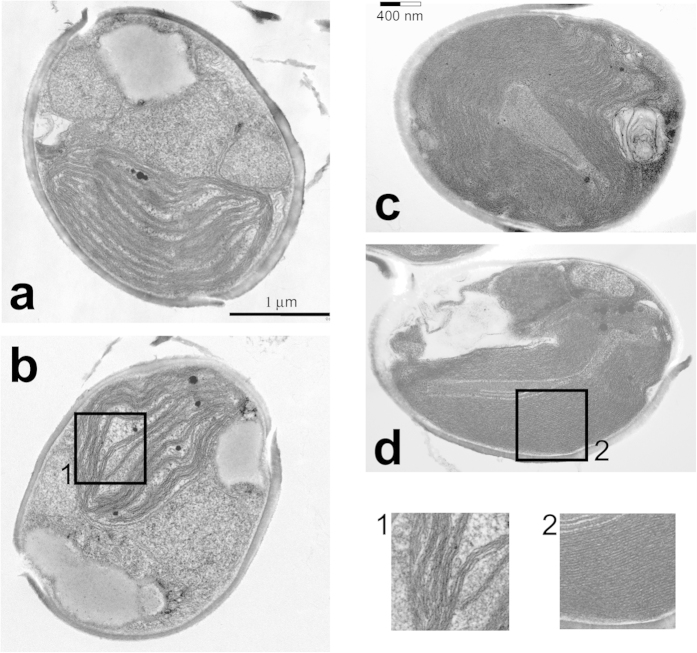
Comparison of ultrastructural changes induced by the prolonged cultivation under red-enhanced illumination in the cells of *P. tricornutum*. (**a**,**b**) – Two representative examples of daylight culture cells; (**c**,**d)** – Examples of the red-light cells; The DL cells show the typical stacked thylakoid membranes grouped by three (box 1). Compare the greatly increased number of homogeneously stacked thylakoids (“superstacks”) in the chloroplast of the RL cells in D, (box 2). To illustrate the size of the PSI-Lhcr membrane patches, such as the one in Fig. 1, a 400 nm scale bar is placed in the panel c.

The observations indicating the segregation of PSI in the RL culture led us directly to the question whether the differences in supramolecular organization of membrane complexes were accompanied with changes in the ultrastructure of the chloroplast membrane system. As seen in Fig. 5, compared to the day-light (DL) culture (Fig. 5a,b), the number of thylakoids was significantly higher in the chloroplasts of the RL culture (c,d). More importantly, while the individual stacks of thylakoids could be resolved in the cells of RL culture (c), these often yielded to large “superstacked” regions containing tens of evenly spaced thylakoids (d). As shown in panel c, the highly stacked regions derived from the regular stacks of three and both forms of membrane organisation coexisted. Considering the current opinions on the organization of complexes in the thylakoid membranes of diatoms and related groups^14^ placing PSI into the unappressed thylakoids, the ultrastructure of the RL culture cells suggest that PSI was excluded from the highly appressed “superstack” regions. For comparison, we added to Fig. 5 a scale bar corresponding to size of the membrane patch shown in Fig. 1.

## Discussion

Despite the general assumption that the high spatial heterogeneity of the thylakoid protein complexes uniquely occurs in plants, our data show that the heterogeneity of photosystems forms in diatom thylakoids under physiologically relevant conditions. Present study demonstrates the segregation of PSI-Lhcr supercomplexes into membrane domains in the secondary plastid of RL-adapted *P. tricornutum*.

Analysis of the electron microscopy images of these membrane regions revealed the spatial arrangement and allowed estimation of the size and shape of the protein complexes present. Calculations indicated tight packing with a mean distance of ~17 nm between particle centres. However, the particles occupying the membrane patches were not organized in highly ordered arrays typical of plant PSII^33^. It should be noted that knowledge on PSI arrangement in the stroma lamella of plant thylakoids is also limited, partly due to the poorly defined surface features of PSI relative to PSII. However, Dekker & Boekema^6^ presented images of a thylakoid membrane patch with aggregated particles, interpreted as PSI complexes, which appear similar to the present observations.

Using a high concentration of α-D-dodecyl-maltoside, Boekema and colleaques^6^,^34^, observed a thylakoid membrane patch with aggregated particles, which they interpreted as an artificial association of PSI complexes. While those aggregated particles might resemble in appearance our present observations, we can safely rule out the possibility of such artificial aggregation in our preparations when considering (i) usage of a very low β-D-dodecyl-maltoside concentration with subsequent multi-step purification, (ii) absence of a similar structure in DL culture analyzed by the same isolation procedure, and (iii) different particle organisation of the membrane sheet.

A deeper analysis of the particle distribution in the RL culture of *P. tricornutum* led to a model of spatial organization of PSI-Lhcr complexes in the membrane based on tight packing of elliptical particles with axes of 14 and 17 nm, i.e. an area of ~190 nm^2^ (Fig. 4b). Taking into account the observed density of one PSI per 220 nm^2^, about 85% of the membrane would be occupied by the PSI-Lhcr particles. The pigment analysis of the patches led to the conclusion that the membranes contained as many as 230 molecules of Chl *a* per PSI complex. Thus, the area density of Chl *a* is more than 1 nm^−2^. This corresponds to the pigment content of PSI core, 96 Chl *a*/90 nm^2^ as reported previously^32^,^35^, and to an estimate of ~200 Chl *a* per 190 nm^2^ (14 × 17 nm) PSI-Lhcr complex.

Using the approximate dimensions of a PSI-Lhcr complex (an elliptical cylinder 14 × 17 × 4.5 nm) and the reported density of pigment-protein complexes^36^ ~0.77 kDa·nm^−3^, one can estimate the mass of the PSI-Lhcr complex to roughly 690 kDa. This is close to the apparent molecular mass 700 kDa of the complex in our CN-PAGE (Fig. 2). Subtracting a mass of 350 kDa, determined for the PSI core (computed from the molar masses of components of the PDB structure 1JB0^30^) from the 690 kDa estimated for the entire complex leaves ~340 kDa for the antenna part. Consequently, a complex of monomeric PSI with two sets of antennas would have a total mass of about 1030 kDa, close to our CN-1050 kDa band. This inferred stoichiometry agrees with previous findings in both red and green algae, where PSI-antenna complexes harbouring an additional row of Lhcr or Lhca proteins, respectively, have been reported^20^,^21^,^22^.

Regarding the protein composition of the PSI antenna in diatoms, the present study is in agreement with previous reports^16^,^19^, except for the absence of Lhcx and Lhcf proteins in our system. Absence of the former can be explained by the overall low-light character of the RL cells, since Lhcx proteins are typically synthesized in response to excess irradiance^37^.

While the difference in antenna protein composition was rather minor, pigment changes were more conspicuous. The PSI-antenna arrays observed in the RL cells exhibited a significantly (~8 fold) reduced Chl *c* content relative to the cited studies. Hence, the extensive modification of pigment composition in RL-adapted cells compared to regular DL culture affects not only the outer light-harvesting complexes of PSII^17^ but likely concerns also the inner, PSI-specific antenna. This degree of plasticity in the pigment composition might also indicate that the requirement of Chl *c* as a ligand in the folding of the Lhcr complexes is not very strict.

Based on our data, large structural domains exclusively containing PSI-Lhcr supercomplexes naturally form in the thylakoid membrane of RL adapted *P. tricornutum*; whereas, this does not occur in DL culture^17^. Present observations warrant a closer examination of the seminal work of Pyszniak & Gibbs^13^ who have used diaminobenzidine staining in thin sections of diatom chloroplasts to show local concentration of PSI. While the authors left room for the possibility that these stained regions were an artefact of the procedure, in light of the present findings it appears that the accumulation of diaminobenzidine might have indeed indicated a certain degree of PSI segregation. However, their putative PSI-rich areas were several times smaller than our regions spanning more than a hundred nanometres. The authors concluded that PSI was present on both the appressed and unappressed membranes but slightly more concentrated on the two outer unappressed membranes of each thylakoid triplet band. One reason why the PSI segregation was not prominent in their study could be the use of cool white fluorescent lamps at 40 μmol photons·m^−2^·s^−1^ for culturing *P. tricornutum* cells. At this point, it should be noted that experimental evidence for a laterally heterogeneous, patch-work like, distribution of photosystem I in thylakoid membranes exists in xanthophytes, another member group of stramenopiles^15^. Since it is not apparent how the illumination conditions of the cultures used in the cited study compare to our case, the question whether the heterogeneity of photosystem distribution is an inherent feature of the species studied or is also under environmental control remains at this point open. Interestingly, the authors also observed changes in protein arrangement, interpreted as antenna redistribution between photosystems, brought about by even a short term change in ambient irradiance. While the latter result does not apply to the present study of a long-term acclimation in *P. tricornutum*, the membrane dynamics in stramenopile thylakoid membranes, which lack the granal/stromal thylakoid division, is an issue deserving attention.

Lepetit and colleagues^14^ presented a comprehensive model for the diatom thylakoid membrane structure and dynamics, where the outer lamellae of the stacked thylakoids are enriched in negatively charged, saturated lipid sulfoquinovosyldiacylglycerol (SQDG) compared to the internal membranes of the stacks that contain relatively higher proportion of neutral monogalactosyldiacylglycerol (MGDG). These differences in lipid distribution result in enrichment of PSI and ATPase in the outer lamellae and PSII-FCP complexes in the inner lamellae of the stacks. The authors proposed that a ratio of the lipids and associated protein content in the membranes was controlled by incident radiation, namely a high-irradiance leading to the expansion of SQDG-rich areas. Adhering to the framework offered by this model, the characteristics of the RL cells can be interpreted as an extreme version of the low-light phenotype of Lepetit and coworkers. That is one in which the SQDG areas harbouring the PSI complexes shrink to patches, causing the observed segregation of the PSI-Lhcr supercomplexes and concomitant loss of the triple-stack morphology of the thylakoid membranes in parts of the chloroplast volume as exhibited by the RL cells in Fig. 5c,d.

This hypothesis leads to a question whether the observed crowding of PSI-Lhcr supercomplexes itself represents a distinct component of the acclimation process or is simply a consequence of a modification of lipid metabolism. The relevance of PSI segregation to the physiology of the *P. tricornutum* cells remains yet to be investigated.

In the present work, association of PSI with the complexes known to play a role in the cyclic electron flow, the NAD(P)H dehydrogenase (NDH) (such as the reported PSI-NDH complex^38^) and cytochrome b_6_/f complex^39^ was not observed. The lack of the latter complex in the PSI-Lhcr regions was not surprising in light of the observations of Yan and coworkers who reported inhibition of the cytochrome b_6_/f complex by SQDG^40^. On the other hand, the PSI-related electron transfer steps are likely to be less adversely affected by macromolecular crowding since the transfer reactions around PSI rely on extramembrane diffusible electron carriers (plastocyanin or cytochrome c_6_, and ferredoxin) as opposed to intramembrane electron transfer from PSII via plastoquinone.

It is possible that the segregation of photosystems and the aggregation of PSI-Lhcr might be needed to prevent energy spillover between photosystems, and to optimize the distribution of excitation energy in a system that utilizes the red-shifted antenna associated with PSII. Detailed analysis of excitation energy flow in the membrane of *P. tricornutum* cells acclimated to different light conditions will be needed to address these possibilities.

## Conclusions

This study provides evidence for the spatial segregation of the photosystems in the secondary plastid of marine diatom *P. tricornutum* grown under red-enhanced light. The large supramolecular assemblies of the PSI-Lhcr complexes containing hundreds of particles span thylakoid membrane areas as large as 0.25 μm across. Presence of these homogenous membrane domains, devoid of other components of the photosynthetic electron flow, indicates a significant structural heterogeneity of the stramenopile thylakoid membrane. In the diatom *P. tricornutum*, the non-random distribution of photosystems may have evolved as a strategy for survival in shaded environment. Our findings show that lateral heterogeneity of photosystems is not an exclusive feature of primary plastids of the green lineage but a more general mechanism operating also in other phototrophic eukaryotes.

## Methods

### Diatom culture and thylakoid membrane processing

Cells of *P. tricornutum* (SAG culture collection, strain 1090-1a) were grown under red-enhanced illumination^17^. Fractionation of the thylakoid membranes and the subsequent purification steps using gel filtration chromatography were performed as described in detail recently^17^.

### Pigment analyses

Pigment composition was analysed using reverse-phase high performance liquid chromatography^17^. Estimate of the P700 content was based on the amplitude of the photo-induced bleaching of the primary donor in the presence of ascorbate/PMS (20 mM/20 μM) using the differential extinction coefficient 64 mM^−1^.cm^−1^ 18,41. The measurements of difference absorption spectra were performed using a locally built kinetic spectrometer^42^,^43^.

### Protein composition analyses

Photosynthetic membrane (super)complexes were separated with a CN-PAGE^44^ on a 4.5–14% linear gradient acrylamide gel. The pigment-protein complexes were visible without gel staining. Apparent molecular masses were estimated by a co-electrophoresis of a high molecular weight protein standard (Novex, Life technologies). Protein identification using mass spectrometry was carried out after the denaturing SDS-PAGE in a second dimension, where the proteins of which the complexes are composed of are separated according to their mass. A low molecular weight protein standard (Fermentas) was used to estimate the protein masses on the gel. MS/MS analysis was performed on a NanoAcquity UPLC (Waters) online coupled to an ESI Q-TOF Premier Mass spectrometer (Waters)^23^. Cytochrome *f* was detected in SDS-PAGE gel by a heme-staining procedure^45^.

### Electron microscopy

Freshly prepared gel filtration fraction of RL-grown culture was used for electron microscopy. The sample was placed on the glow-discharged carbon-coated copper grids and negatively stained with 1.5% uranyl acetate. Microscopy images were taken with JEOL 1010 transmission electron microscope (JEOL, Japan) equipped with CCD Sis MegaView III camera, using 80 kV at 120000x  magnification.

Harvested cells of RL- and DL-grown cultures were fixed overnight with 2.5% glutaraldehyde in phosphate buffer at 4 °C, post-fixed in 1% OsO_4_ in phosphate buffer for 2 h and embedded in 2% agarose. The segments of agarose containing cells were dehydrated in isopropyl alcohol series, embedded in Spurr’s epoxy resin. Ultrathin sections were cut with a UCT Ultramicrotome (Leica) using a diamond knife and post-stained with 5% aqueous uranyl acetate followed by lead citrate. The specimens were examined with JEOL 1010 electron microscope.

### Image analysis

The coordinates of particles in the membrane patches were extracted from the images using the following procedure. The images were bandpass filtered (3 to 20 pixels) to enhance contrast and convolved with a Gaussian function of FWHM of 7 nm. Positions of particles were then obtained as local maxima on the resulting smooth surface. The search procedure was automatic. Occasional (<1% particles found) particle overlaps (peak-to-peak distance < FWHM) were resolved manually.

The pair-correlation function *g*(*r*) was calculated as described previously^46^. For a particle in the two-dimensional distribution it relates to the probability of finding another particle within an annulus of radius *r* and a small thickness *dr*. The number of particles found at given *r* is normalized using the mean particle density and the annulus area, 2π*rdr*, so that for randomly distributed particles the pair correlation function assumes a constant value of 1. The total number of analysed particles in our samples was over 1500. The *dr* for *g*(*r*) computation was chosen equal to one image pixel, i.e. 0.64 nm. The maximum search radius, *r*_max_, for each complex was set to the distance to the closest membrane border^47^. All computations were performed to the precision of one image pixel.

### Computer simulations

Simulation was used to test the dimensions and packing of particles occupying the *P. tricornutum* membranes that could give rise of the observed pair-correlation function. This was accomplished by simulated diffusion^48^ of an ensemble of elliptical discs within a square enclosure of an area corresponding to the number of particles × 220 nm^2^. Hard, non-interacting particles were assumed. The run was initiated from tightly packed, aligned particles. The particles performed a random walk over a square lattice in steps of 0.5 nm (diagonal steps were not allowed). Rotation about vertical axis of particles was also allowed in random steps chosen from the interval 〈−10, 10〉 degrees. Progress of a sample simulation is shown in Supplementary Fig. S5. After the initial quick dispersion of particles over the enclosure, duration of the run determined the amount of disorder of the particle orientations. In order that the modelled distribution of particles could be meaningfully compared to the experimental observation, the error of determination of position of the observed particles in the images had to be estimated. To this end, we utilized images of trimeric cyanobacterial PSI particles, since the structure of the isolated PSI trimers could be resolved easily using the same staining procedure and microscope settings as that used for the membrane patches (Supplementary Fig. S6). Moreover, due to the rotational symmetry of the cyanobacterial particle, geometrical centres of individual PSI RCs could be easily determined. Individual images of PSI trimers were selected, aligned and averaged and the average image was used to determine the coordinates of PSI RCs with respect to the centre of the trimer. The individual aligned images of trimers, from which the average was computed, were put into a single image file and the same search procedure as used for the membrane patches was applied, obtaining the coordinates of PSI RCs. The difference in positions of PSI RCs found using the search procedure in the source images of trimers were compared to the coordinates obtained from the averaged image. The estimated error was found to be approximately normally distributed with the standard deviation of 2 nm. Random values drawn from this distribution were then applied to the coordinates of the simulated particles.

## Additional Information

**How to cite this article**: Bína, D. *et al.* Novel structural aspect of the diatom thylakoid membrane: lateral segregation of photosystem I under red-enhanced illumination. *Sci. Rep.* **6**, 25583; doi: 10.1038/srep25583 (2016).

## Supplementary Material

Supplementary Information

## Figures and Tables

**Table 1:** Pigment composition of PSI-rich membrane rafts purified from thylakoids of red-light adapted cells of *P. tricornutum.*

Chl *a*	Chl *c*	Fucoxanthin	Diadinoxanthin	β-carotene	
1	0.012 ± 0.002	0.144 ± 0.003	0.118 ± 0.007	0.097 ± 0.008	1/Chl *a*
230	3	33	27	22	22/β-car^a^
233	3	34	27	23	1/P700^b^

Pigment ratios are expressed per Chl *a*, per β-carotene and per P700 (primary donor of PSI) for convenience. Mean ± SD from three replicates is shown.

^a^Based on X-ray structure (1JB0) showing 22 β-carotene molecules in the PSI core and using only the HPLC data.

^b^Based on a value of 233 ± 14 Chl *a* per one P700 obtained using light-induced oxidation of PSI RC.
