# Supplementary material for: Novel structural aspect of the diatom thylakoid membrane: lateral segregation of photosystem I under red-enhanced illumination
Source: Sci Rep. 2016 May 5;6:25583. doi: 10.1038/srep25583 (PMC4857733; doi:10.1038/srep25583)
Supplement: Supplementary Information [file srep25583-s1.pdf]

Supplementary information for

Novel structural aspect of the diatom thylakoid membrane: lateral segregation of photosystem I under red-enhanced illumination

David Bína, Miroslava Herbstová, Zdenko Gardian, František Vácha & Radek Litvín

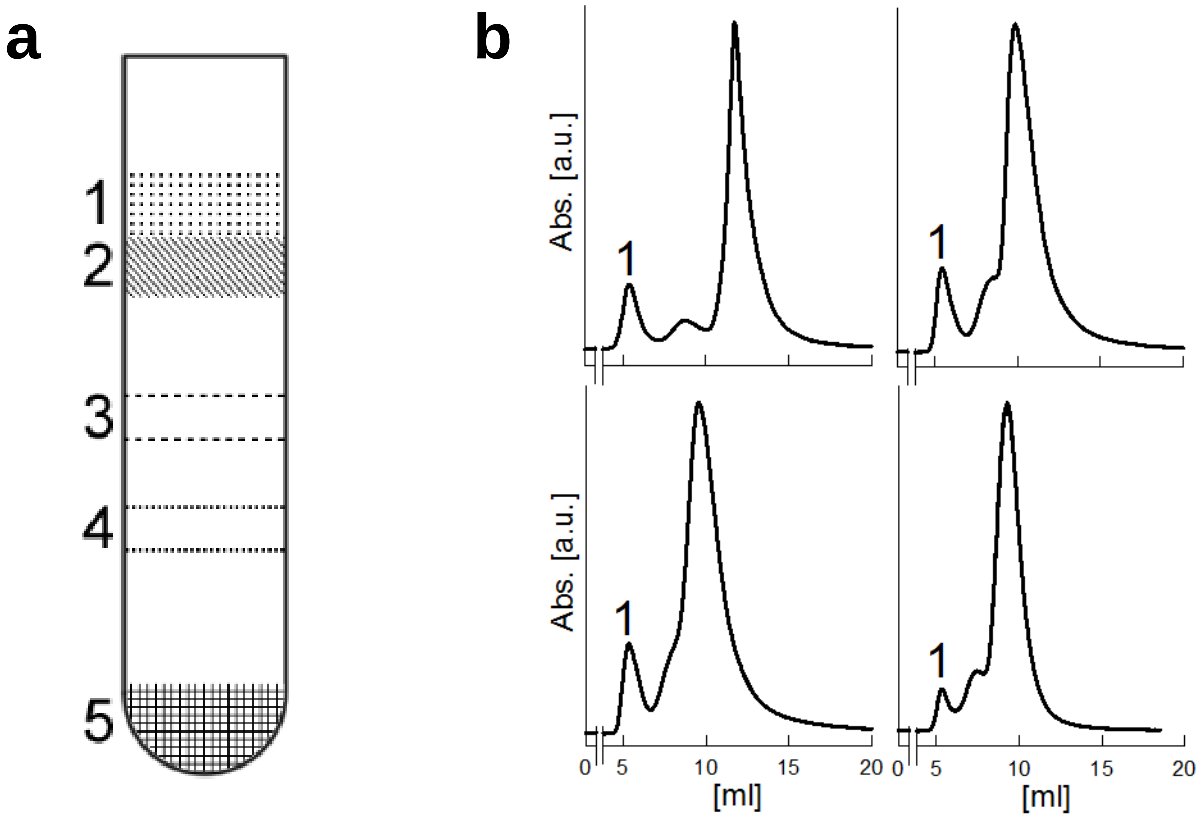

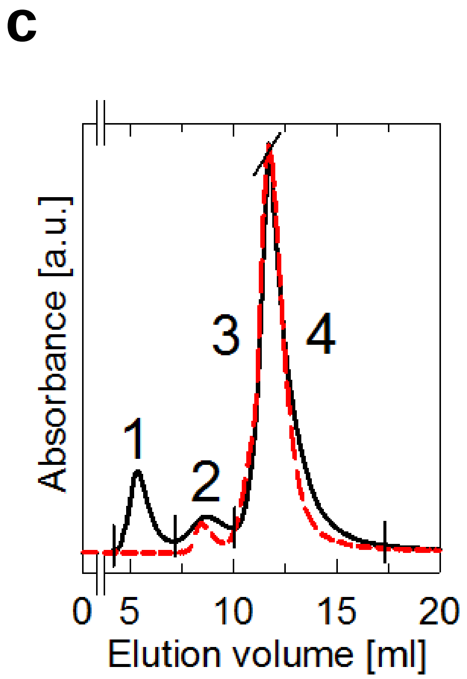

Supplementary Figure S1 a sucrose density gradient from RL grown *P. tricornutum.* **b** Examples of the gel filtration chromatograms of the sucrose gradient zone 5. The membrane patches were found in the first fraction (1); **c** comparison of the gel filtration chromatograms of sucrose gradient zone 5 from RL- (black solid line) and day-light (DL)-grown *P. tricornutum* (red dashed line). The superposed elution profiles show that fraction 1 containing PSI-rich membrane rafts is missing in DL sample.

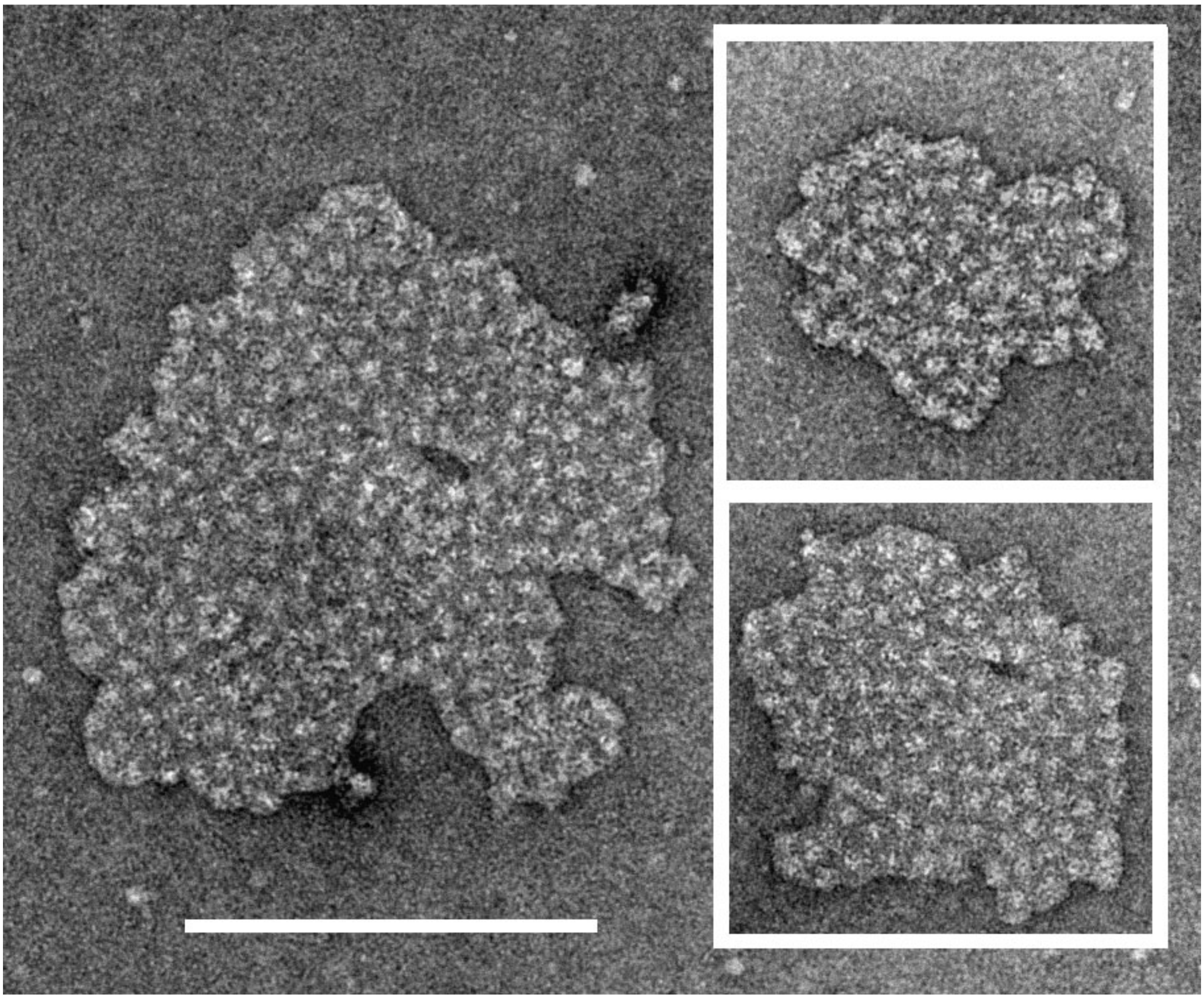

Supplementary Figure S2 Examples of the negatively stained membrane patches isolated from *P. tricornutum* grown under red-enhanced illumination. Scale bar corresponds to 200 nm.

Supplementary Table 1a Light harvesting proteins found in the association with PSI identified by MS/MS analysis of the marked bands in Fig. 2. Data are based on the three biological replicates.

Band	ID Name	AA	Peptides	Start	End	Products[1]	MH+Error (Da)[2]	Score
	EEC51848.1							
1, a	**Lhcr4**	215	(K)GYVGDAGFDPFR(F)	59	70	20	-0,0048	4740,802
1, a			(R)VVYPLPEGYEGLTSVTAHDALVK(Q)	107	128	18	-0,012	671,3947
1, a			(R)FSDFLPMDFLR(E)	71	81	17	-0,0052	4081,051
1, a			(R)RPGDFGFDPAGFLK(N)	160	173	10	-0,0109	72,9889
1, a			(K)YVASESLPFMEYPPNLK(G)	42	58	3	0,0073	3,6603
	EEC44790.1							
1, a	**Lhcr2**		(R)APGDFGLDPLK(I)	157	167	14	-0,0035	900,8351
	EEC46230.1							
2	**Lhcr13**	206	(K)TEAGNLNFDYLGLK(K)	152	165	16	0,0002	141,5039
2			(K)AFDTFPNMFPDEQYLR(D)	53	68	7	0,009	161,2747
	EEC50289.1							
3, c	**Lhcr1**	200	(K)VTMEDMFSDSSR(E)	135	146	7	-0,0038	116,5002
3, c			(K)SMPFLTAPK(N)	36	44	6	-0,004	69,4386
3, c			(K)NTGGYVGDVGFDPLGFSDNFDMK(W)	45	67	5	0,0115	0,6790
	EEC50833.1							
3,4,c	**Lhcr3**	199	(K)HYGEGEPGDLGFDGGQLK(G)	142	159	11	-0,015	39,8421
3,4,c			(K)SIPFLVKPDK(L)	40	49	5	-0,004	2117,374
3,4,c			(R)LSDIQTDLK(Y))	65	73	9	-0,0076	49,9017
3,4,c			(L)GFDGGQLK(G)	152	159	0	0,0046	0
	EEC51320.1							
3,4,b,c	**Lhcr12**	202	(R)EPGYFGFGTK(Y)	148	157	11	-0,0065	73,7076
3,4,b,c			(K)SPAMPFLPYPENLK(G)	35	48	11	-0,0017	1008,342
3,4,b.c			(K)GFPYF(-)	198	202	3	0,0029	121,7254
3,4,b,c			(K)GYIGDDIGFDPLGFSDYFPMDYLR(E)	49	72	5	-0,0039	16,8937
	EEC46063.1							
4,b,c	**Lhcr14**	198	(K)LAETYEPFLPWFR(E)	55	67	24	-0,0015	4438,578
4,b,c			(R)IPGEAYSFAAIPK(T)	92	104	16	-0,0051	228,7691
4,b,c			(K)TIDAHDALLK(S)	105	114	13	-0,0052	2350,263
4,b,c			(R)TPGDFGWTLMAPK(D)	146	158	11	-0,0052	342,6516
4,b,c			(K)FDPLK(L)	50	54	1	-0,0005	11,344
	ABK20584.11							
3,4,d	**PsaF**	185	(K)NPAESEIIINVPLAIK(I)	137	152	24	0,0028	487,6358
3,4,d			(R)MSQYQADSPPSLALQQQIDR(T)	53	72	9	-0,0212	68,8318
3,4,d			(K)IMTTGYIWPISAWQELISGELIAPK(D)	153	177			
	ABK20584.1							
5, e	**PsaD**	139	(K)EQIFEMPIGGAAIMR(S)	38	52	19	0,0026	3384,881

5, e			(R)SGENLLYLAR(K)	53	62	18	-0,0034	1587,216
5, e			(K)FSGINTYES(-)	131	139	17	-0,0029	179,2637
5, e			(K)YAITWTSPK(E)	29	37	10	-0,0031	442,8219
5, e			(R)IFPSGEVQYLHPK(D)	86	98	8	-0,0146	85,2302
5, e			(R)DFSIGKNPNPASIK(F)	117	130	7	0,0046	247,3105
5, e			(F)PSGEVQYLHPK(D)	88	98	6	-0,0038	3,0793
5, e			(R)DFSIGK(N)	117	122	3	-0,0021	6,1579
	YP_874366.1							
6, f	**PsaL**	151	(R)AILQNLPAYR(F)	29	38	16	-0,0052	2759,265
6, f			(M)ANFIKPYNDDPFVGHLATPITSSAVTR(A)	2	28	9	-0,0337	9,4111
6, f			(R)FGLTPLLR(G)	39	46	12	-0,0063	1534,687
	A0T0F3.1							
g	**PsaE**	69	(K)VNYSGTNTNNFALSELVEVSSPK(K)	43	65	10	-0,0151	1,7448

[1]Number of identified CID (collision induced dissociation) fragment per peptide.
[2]Measurement error (Da). The number indicates the deviation from expected (calculated) value.

Supplementary Table 1b Molecular weights (MW), estimated from gene sequences, of Lhcr antenna proteins and small PSI subunits identified in the PSI aggregates.

Protein	MW (Da)
Lhcr1	21 517
Lhcr2	23 383
Lhcr3	21 093
Lhcr4	23 223
Lhcr12	21 694
Lhcr13	22 252
Lhcr14	21 321
PsaF	20 543
PsaL	16 125
PsaD	15 556
PsaE	7 851

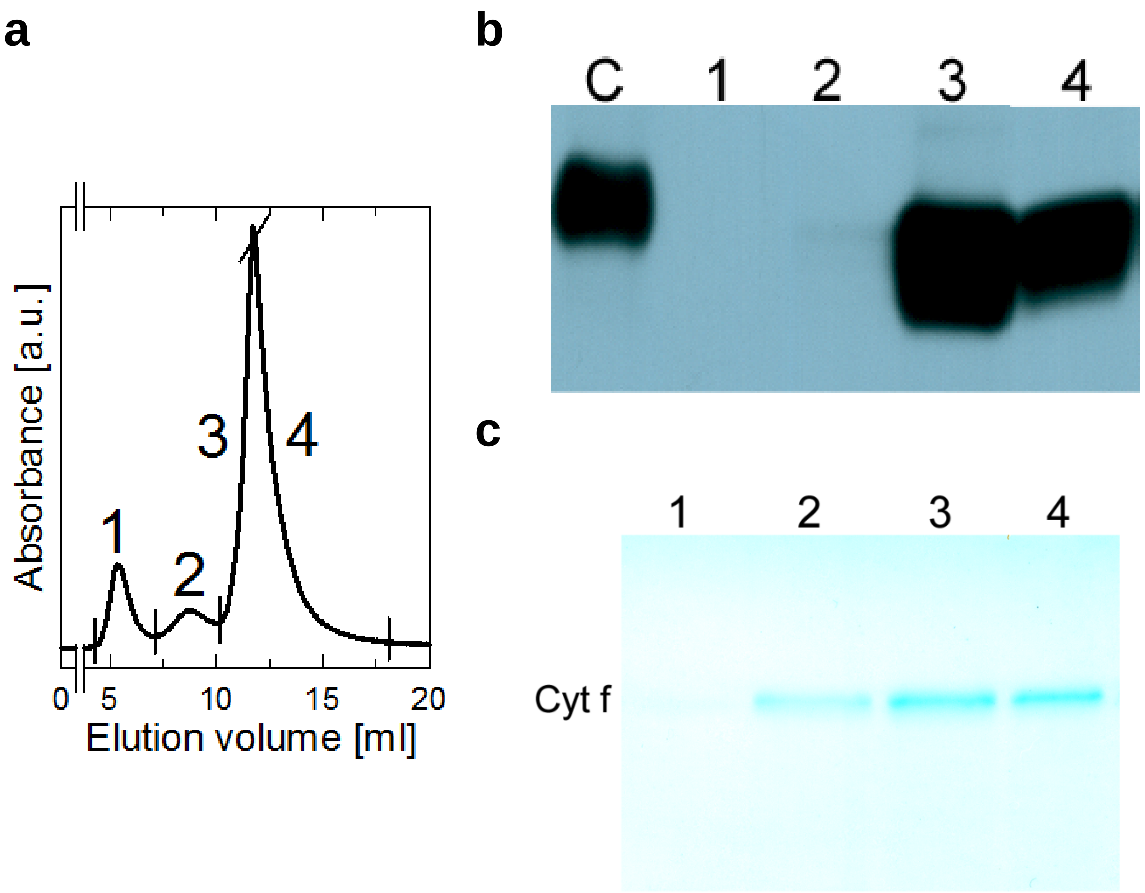

Supplementary Figure S3 Biochemical analysis detecting the photosynthetic complexes in gel filtration fractions from RL-grown *P. tricornutum*. **(a)** A representative chromatography profile of the sucrose density gradient zone 5. **(b)** Western Blot detection of PsbA (D1 protein) of photosystem II in fractions 1 - 4. A total chlorophyll amount of 2 µg was loaded per well and separated on a 14% precast gel (Serva), electroblotted onto PVDF membrane, and detected using rabbit polyclonal antibody (AS05 084, Agrisera, Sweden). “C” stands for PsbA positive control (Agrisera, Sweden). **(c)** Detection of heme-containing proteins in SDS-PAGE gel after TMBZ peroxidase activity staining.

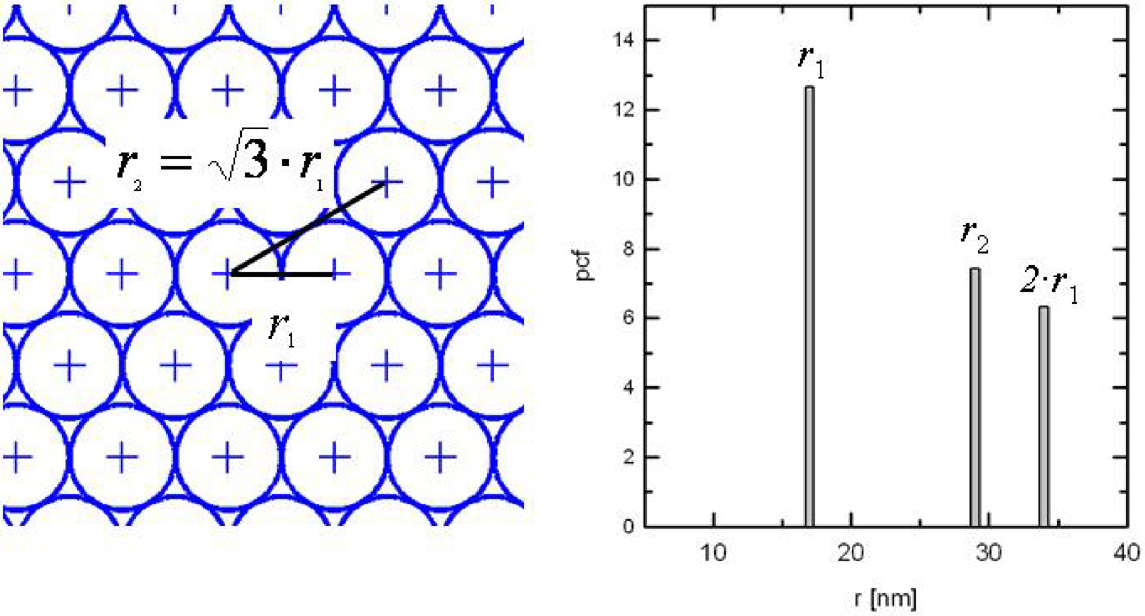

Supplementary Figure S4 Illustration of a point-correlation function for a perfect hexagonal packing of circles with a center spacing of r_1, equal to circle diameter.

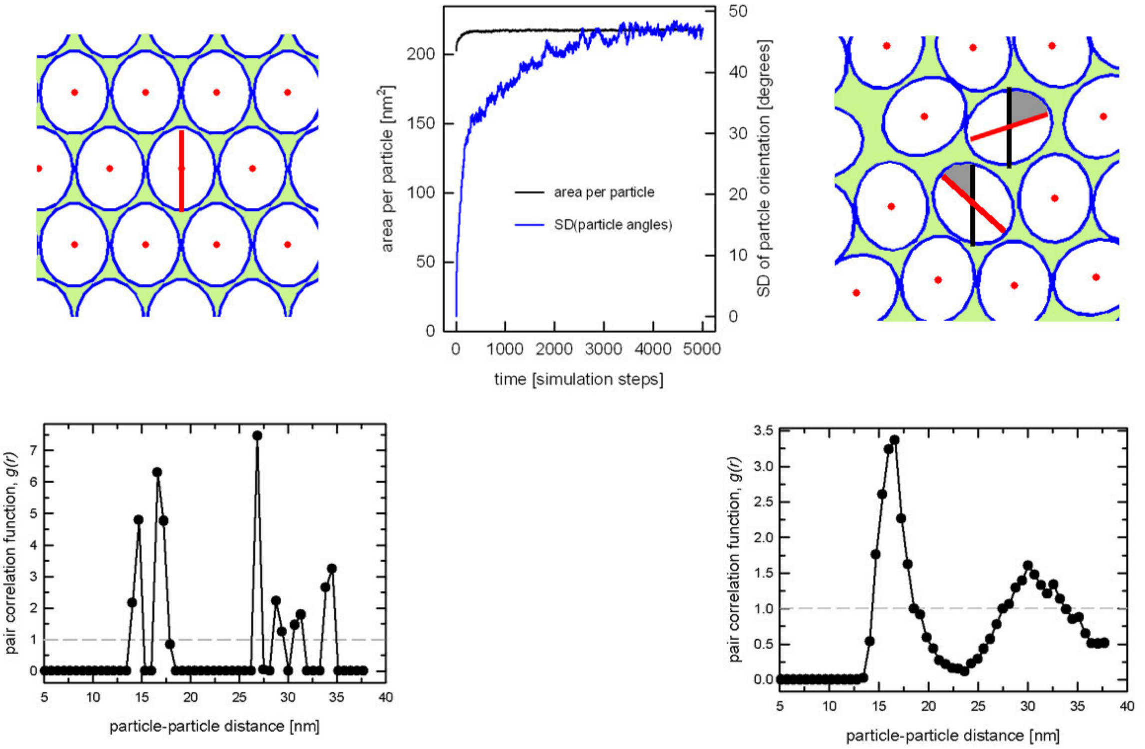

Supplementary Figure S5 Example of a course of a simulation of packing of PSI-Lhcr particles modeled as hard ellipses 14 × 17 nm. Simulation started from the particles oriented as shown in the left panel (density of 1 particle per 200 nm^2) and proceeded to the situation illustrated in the right panel (final density of 1 / 220 nm^2). The middle panel shows the increase of disorder (blue), quantified by the standard deviation (SD) of particle angles laying in the interval ⟨-90,+90⟩ degrees with respect to the vertical axis, as shown by the grey areas in the right panel. The red dots denote the geometrical centers of the ellipses. The graphs in the lower part of the figure show the corresponding pair-correlation functions (pcf) of given particle distributions. After the simulation run, the centers were shifted in x and y direction by random numbers drawn from a normal distribution with SD = 2 nm (see Supplemental Fig. S6) to mimic the error in center determination in the electron micrographs. For the corresponding final pcf see Fig. 4 in the main text.

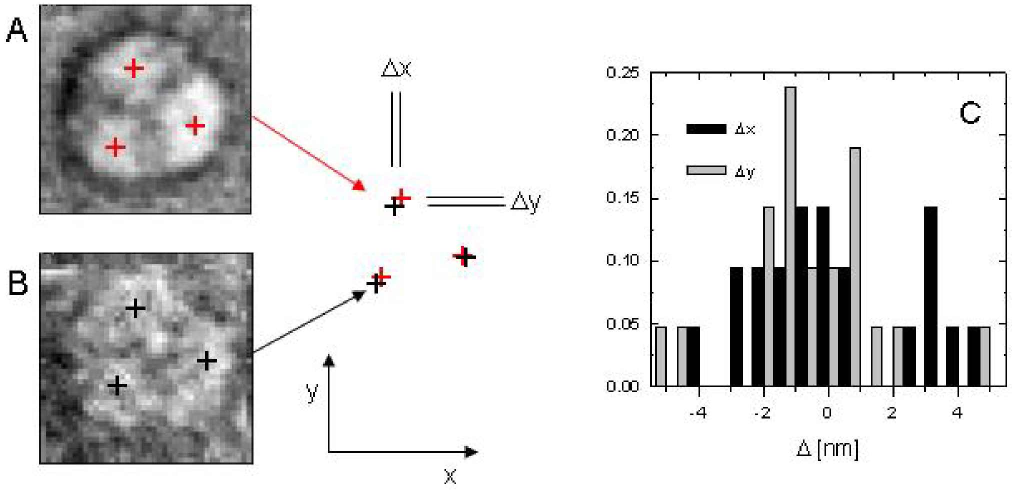

Supplementary Figure S6 Illustration of the approach used for the estimation of the error of determination of particle position from negatively stained images using the cyanobacterial PSI trimer. **A** the average image of PSI trimer; **B** example of a raw particle image, aligned with A; **C** histogram of relative positions of 48 trimer subunits respective to the average shown in panel A. The microscope settings and the staining method were the same as for the membrane patches, as described in Methods.
